# Are embryonic stem cell markers and ALDH1A1 relevant in the context of breast cancer estrogen positivity?

**DOI:** 10.1002/cam4.7004

**Published:** 2024-02-24

**Authors:** Noha A. Mousa, Amal Hussein, Noha M. Elemam, Ghada Mohammed, Mona Elwany, Tasneem Basha, Amal A. AlHammadi, Rana S. Majzob, Iman M. Talaat

**Affiliations:** ^1^ Clinical Sciences Department, College of Medicine University of Sharjah Sharjah United Arab Emirates; ^2^ Family and Community Medicine and Behavioural Sciences Department, College of Medicine University of Sharjah Sharjah United Arab Emirates; ^3^ Research Institute for Medical and Health Sciences, University of Sharjah Sharjah United Arab Emirates; ^4^ Medical Research Institute, Alexandria University Alexandria Egypt; ^5^ Pathology Department, Faculty of Medicine Alexandria University Alexandria Egypt

**Keywords:** ALDH1A1, cancer stem cells, embryonic, ER‐positive breast cancer, pluripotency markers

## Abstract

**Background:**

Embryonic pluripotency markers are recognized for their role in ER− BC aggressiveness, but their significance in ER+ BC remains unclear. This study aims to investigate the prevalence of expression of pluripotency markers in ER+ BC and their effect on survival and prognostic indicators.

**Methods:**

We analyzed data of ER+ BC patients from three large cancer datasets to assess the expression of three pluripotency markers (NANOG, SOX‐2, and OCT4), and the stem cell marker ALDH1A1. Additionally, we investigated associations between gene expression, through mRNA‐Seq analysis, and overall survival (OS). The prevalence of mutational variants within these genes was explored. Using immunohistochemistry (IHC), we examined the expression and associations with clinicopathologic prognostic indicators of the four markers in 81 ER+ BC patients.

**Results:**

Through computational analysis, NANOG and ALDH1A1 genes were significantly upregulated in ER+ BC compared to ER‐ BC patients (*p* < 0.001), while POU5F1 (OCT4) was downregulated (*p* < 0.001). NANOG showed an adverse impact on OS whereas ALDH1A1 was associated with a highly significant improved survival in ER+ BC (*p* = 4.7e‐6), except for the PR− and HER2+ subgroups. Copy number alterations (CNAs) ranged from 0.4% to 1.6% in these genes, with the highest rate detected in SOX2. In the IHC study, approximately one‐third of tumors showed moderate to strong expression of each of the four markers, with 2–4 markers strongly co‐expressed in 56.8% of cases. OCT‐4 and ALDH1A1 showed a significant association with a high KI‐67 index (*p* = 0.009 and 0.008, respectively), while SOX2 showed a significant association with perinodal fat invasion (*p* = 0.017).

**Conclusion:**

Pluripotency markers and ALDH1A1 are substantially expressed in ER+ BC tumors with different, yet significant, associations with prognostic and survival outcomes. This study suggests these markers as targets for prospective clinical validation studies of their prognostic value and their possible therapeutic roles.

## Graphical abstract

In this study, we present evidence regarding the role of the three master pluripotency markers (NANOG, SOX2 and OCT4) along with the stem cell marker ALDH1A1 in estrogen receptor positive BC tumors. The findings support that cancer stem cell markers are considerably expressed in ER+ BC and have variable, yet significant, associations with each other and with key clinicopathologic prognostic indicators, such as Ki‐67, Progesterone receptor, and perinodal fat invasion. Survival analysis showed significant effect of these markers on survival of ER+ BC patients.

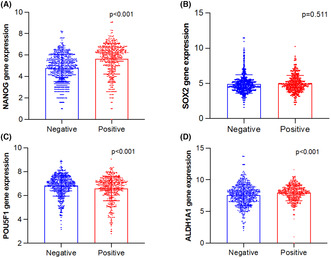

## 1 INTRODUCTION

Estrogen, the primary female sex hormone, plays a central role in breast cancer (BC) pathogenesis.^1^, ^2^, ^3^, ^4^ Estrogen Receptor‐positive breast cancer (ER+ BC), constituting approximately 70% of all BC cases, is marked by the nuclear expression of the ER.^5^, ^6^, ^7^ As such, these tumors are considered estrogen‐dependent/estrogen‐sensitive, and the mainstay treatment approach includes tumor excision followed by anti‐estrogen or estrogen deprivation therapies (i.e., endocrine therapies).^6^ However, a substantial number of patients either have de novo resistance or develop acquired resistance to these therapies, posing a challenge in understanding resistance causes and identifying at‐risk patients.^8^

In ER+ BC, the role of breast cancer stem cells (CSCs) in prognosis and resistance to endocrine therapies has received less attention compared to ER‐negative breast cancer (ER‐ BC). This may be attributed to the general perception of ER+ BC tumors as well‐differentiated and hormonally responsive, leading to an assumption that CSCs play a limited role in these tumors. However, recent studies have challenged this assumption.^9^, ^10^, ^11^, ^12^ Among these, the findings by Simoes et al. that the overexpression of the stem cell marker ALDH1 could predict the failure of tamoxifen therapy.^10^

Additionally, other studies highlighted the role of stem cells in tamoxifen resistance.^9^

As initially identified in human embryonic stem cells, pluripotency markers are essential for inducing and maintaining cellular stemness, which encompasses long‐term self‐renewal, plasticity, and undifferentiated cell state. The inherent expression or the experimental induction of pluripotency markers has been increasingly linked to enhanced CSC properties and the activation of drug resistance mechanisms.^13^, ^14^, ^15^ In a previous in vitro investigation, patient‐derived BC cells isolated from an ER+ tumor exhibited CSC‐like properties and, displayed high expression of pluripotency markers and ALDH1A1, rendering them resistant to hormonal interventions as well as antihormonal medications.^16^

In this study, we aim to investigate the expression patterns of key pluripotency and stem cell markers in ER + BC tumors and their associations with well‐established clinicopathologic prognostic markers. While several markers play regulatory roles in stem cells, however, in this investigation we focus on the three master embryonic pluripotency markers: NANOG, sex‐determining region Y‐box 2 (SOX2), and octamer‐binding transcription factor 4 (OCT4). The three transcription factors co‐regulate self‐renewal and pluripotency in embryonic stem cells, with evidence linking them to poor prognosis in various cancers, including BC.^17^, ^18^, ^19^, ^20^ In addition, we explore the stem cell marker aldehyde dehydrogenase 1 family member A1 (ALDH1A1) due to its recognized role in stemness and therapeutic resistance in several cancers.

## 2 METHODS

### 2.1 In silico studies of pluripotency genes and ALDH1A1 in ER+ BC patients and their associations with survival outcomes

We examined the expression of NANOG, SOX2, POU5F1 (the gene encoding OCT4), and ALDH1A1 in a cohort of estrogen receptor‐positive (ER+ BC) patients (*n* = 532) in comparison to ER‐negative (ER‐ BC) patients (*N* = 729) using the GENT2 tool (http://gent2.appex.kr/gent2/).^21^ This is a publicly available platform that enables the analysis of gene expression patterns in normal and tumor tissues. The resulting data were visualized using GraphPad Prism 9 (GraphPad, Boston, MA, USA).

Subsequently, Kaplan–Meier survival curves were derived from RNA sequencing data for ER+ BC patients using the KM plotter (https://kmplot.com/analysis).^22^ This is an extensive database with 30 k + samples of different cancers, which is mainly used for the discovery and validation of survival biomarkers. To enhance relevance to our specific study objective consistency with the profile of our clinical study population, we specifically chose ER+ BC cases that received endocrine therapy for inclusion in survival analysis (*n* = 2279). Subgroup survival analyses were conducted for ER+ progesterone receptor‐positive (PR+) BC patients (*n* = 2040) and ER+ progesterone receptor‐negative (PR−) BC patients (*n* = 119). Additionally, analysis within the subgroup of ER + BC with HER2+ expression was explored (*n* = 252).

Additionally, we investigated the prevalence and characterization of copy number alterations (CNAs) and mutational variations of the four genes of interest using the cBioPortal repository (https://www.cbioportal.org/). This is an open‐source, open‐access resource encompassing multidimensional cancer genomics datasets from thousands of patients. In this search, we applied the filter https://bit.ly/47jPOJf to select ER‐positive primary BC tumors (*n* = 2607).

### 2.2 Immunohistochemistry (IHC) study design and subjects

This was a retrospective cohort study that aimed: First, to assess the expression status of the three pluripotency markers and ALDH1A1 in formalin‐fixed paraffin‐embedded (FFPE) of a selected group of ER+ BC patients. Second, to verify if there is any association between the level of the expression of these markers and one or more of the clinically recognized prognostic markers. Institutional ethical approval was obtained for the immunohistochemistry (IHC) study (REC‐20‐02‐03‐01). FFPE histopathologic sections were selected from tumor tissue obtained at primary surgery for BC patients, who were identified as ER+. ER‐positivity was defined ≥1% expression as per the clinical guidelines.^23^ Tumor sections of patients with metastatic BC were excluded. Included tumor sections were assessed histologically according to the 2019 WHO classification,^24^ and the staging was performed according to the TNM staging (8th edition).^25^

#### 2.2.1 IHC methods

The IHC for the following primary antibodies: anti‐NANOG, anti‐OCT4, anti‐SOX2, and anti‐ALDH1A1, was performed manually on 4‐μm‐thick sections as previously described.^26^ In brief, FFPE sections were deparaffinized in xylene, rehydrated in a series of ethanol, immersed in 0.01 M citrate buffer (pH 6.0), and heated in a microwave oven at full power for 2–5 min, then left in buffer to cool at room temperature. The sections were incubated in 0.3% hydrogen peroxide for 20 min to block endogenous peroxidase activity. Incubation with the primary antibodies (Anti‐NANOG [ab62734], anti‐OCT4 [ab194076], anti‐SOX2 [ab93689], and anti‐ALDH1A1 [ab215996]; Abcam, Cambridge, UK) at a concentration of 1:200, diluted in 1% bovine serum albumin/tris‐buffered saline was carried out overnight in a humid chamber at 4°C according to the manufacturer's instructions. On the following day, the slides were washed with PBS and incubated with biotinylated secondary antibody (SignalStain® Boost IHC Detection Reagent; Cell Signaling Technology) for 30 min at 20°C, then with avidin‐biotin‐peroxidase complex for 30 min at 20°C (Vectastain ABC kit; Abcam, Cambridge, UK). For visualization, the peroxidase/DAB DAKO Real ENVision detection system (DAKO, Glostrup, Denmark) was used, following the manufacturer's instructions. For each run of IHC staining, positive and negative control sections were included to confirm the primary antibodies' specificity.

#### 2.2.2 Immunohistochemical studies interpretation

Immunopositivity of the four primary antibodies (NANOG, OCT4, SOX2, and ALDH1A1) was assessed for each patient. The evaluator was blinded to the clinical data and the patients' outcomes of the corresponding samples. The whole tissue section was examined in two independent examinations using an Olympus microscope (BX51; Olympus, Tokyo, Japan). The percentage of positively stained tumor cells (PP) and the staining intensity (SI) were determined to calculate the immunoreactive score (IRS): IRS = SI × PP as previously described.^27^ The scores for the percentage of the positively stained cells were assigned as follows: score 0: <10% positive cells, score 1: 10%–50% positive cells, score 2: 51%–75% positive cells, and score 3: >75% positive cells.^28^ Regarding the SI: (0) no staining; (1) weakly positive; (2) moderately positive; and (3) strongly positive. The IRS score ranging from 0 to 3 was designated as negative, a score of 4 or 5 as mildly positive, 6 or 7 as moderately positive, and 8 or 9 as strongly positive. In this study, negative and mild expressions of each marker were considered one group, whereas the moderate and strong expressions were considered another group.

#### 2.2.3 Statistical analysis

For the IHC study, data were first compiled, coded, and entered on Microsoft Excel (©Microsoft Inc., Redmond, Seattle, version 2016). They were then imported to SPSS for analysis (IBM SPSS Statistics for Windows, Released 2021. Version 28. Armonk, NY: IBM Corp). Descriptive univariate analysis was performed as appropriate to the type of data. Frequencies and relative frequencies were reported for categorical variables, while for scale variables, means and standard deviations were reported for normally distributed data, and medians and interquartile range (IQR) were reported for skewed data. Histograms and Q–Q plots were first used to visually check for the normality of continuous data. Kolmogorov–Smirnov test was used to statistically test the normality of data. In this study, the Pearson chi‐squared test was used to test the association between categorical variables, and Fisher's exact test was reported for associations showing low expected counts in more than 20% of the cells of cross‐tabulations. In this study, the level of significance was set at 0.05.

## 3 RESULTS

### 3.1 Computational studies outcomes

#### 3.1.1 Gene expression analysis of NANOG, SOX2, POU5F1 (OCT4 gene) and ALDH1A1 in relation to estrogen receptor status

**FIGURE 1:**
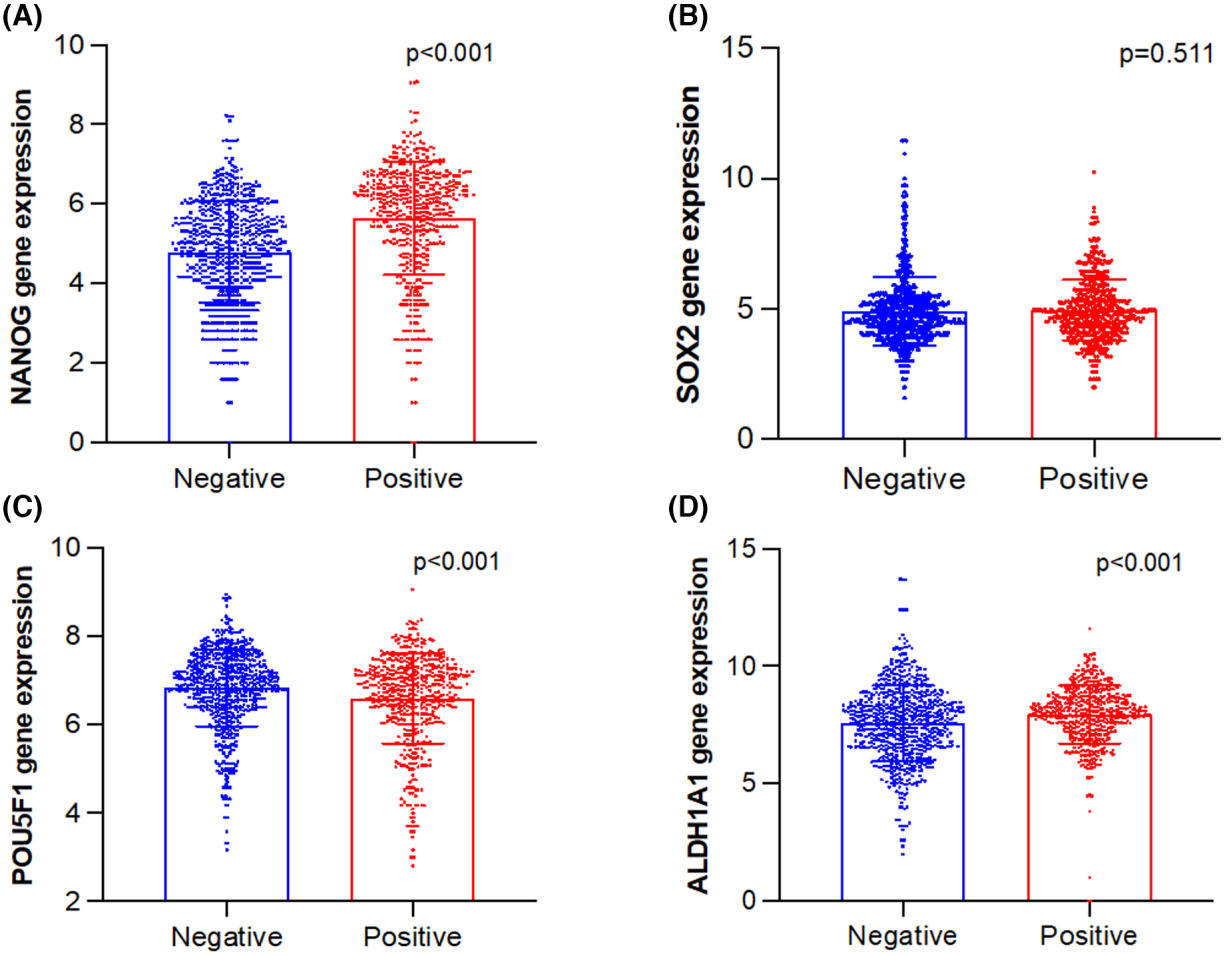
Expression of NANOG (A), SOX2 (B), POU5F1(C), and ALDH1A1(D) in ER+ BC patients (red for ER+ BC *n* = 532, blue for ER‐BC *n* = 729). Data are generated using Gent2 tool (http://gent2.appex.kr/gent2).

Using publicly available datasets as detailed above, we identified a statistically significant upregulation in the expression of the NANOG and ALDH1A1 genes in ER+ BC patients compared to ER‐ BC patients (*p* < 0.001). Conversely, the POU5F1 gene showed significant downregulation in ER+ BC patients (*p* < 0.001), as shown in Figure 1.

#### 3.1.2 Survival outcomes as by RNA‐Seq gene expression of the pluripotency markers and ALDH1A1 in ER+ BC patients

**FIGURE 2:**
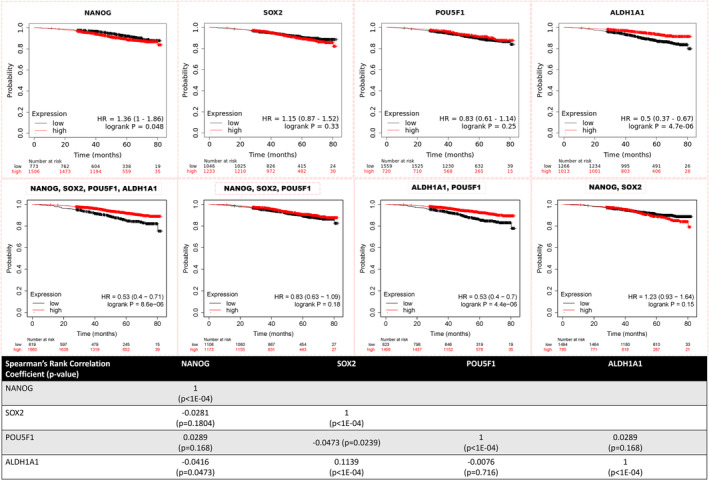
Kaplan–Meier graph showing overall survival (OS) analysis of women with ER+ BC in relation to the RNA‐Seq expression of NANOG, SOX2, POU5F1, and ALDH1A1 (independently and combined analysis of one or more of the four genes). Correlation analysis using Spearman's correlation for the expression of the four markers is shown in the lower table. Data are generated from the KM plotter (https://kmplot.com/analysis/) for ER+ BC patients who received endocrine therapies (*n* = 2279).

In this analysis utilizing the KM Plotter, we examined the effect of the three pluripotency genes and ALDH1A1 expression on the overall survival (OS) of women diagnosed with ER+ BC. We analyzed OS data for a cohort of 2279 women who all received anti‐estrogen endocrine therapy, with follow‐up data available for up to 80 months. As illustrated in Figure 2, high NANOG gene expression was associated with reduced OS (HR = 1.36, 95% CI: 1.00–1.86, *p*‐value = 0.0479). In contrast, elevated ALDH1A1 expression was linked to a statistically significant improvement in the survival of ER+ BC (HR = 0.50, 95% CI: 0.37–0.67, *p*‐value = 4.7e‐6). Utilizing mean gene expression data from various combinations of the four genes highlighted the consistently significant favorable impact of ALDH1A1 on survival outcomes.

**FIGURE 3:**
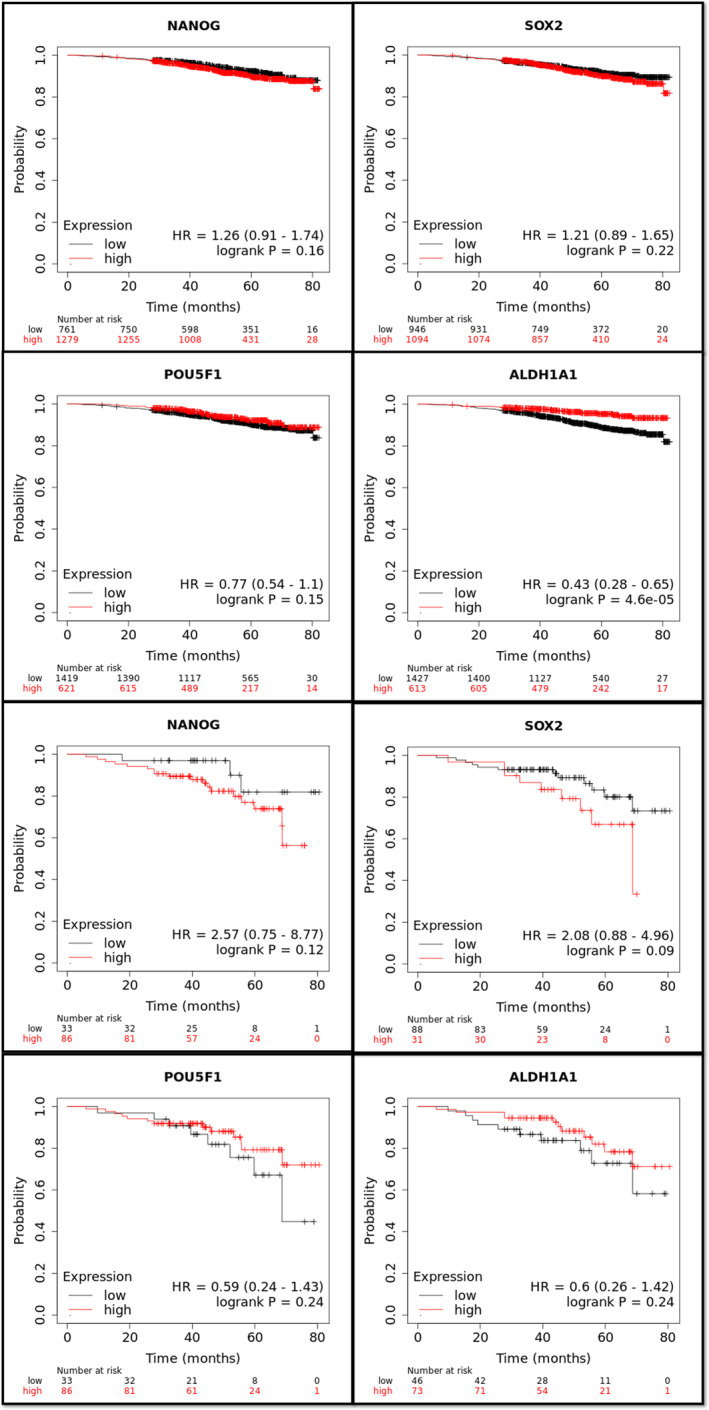
Kaplan–Meier survival analysis showing the effect of PR status on overall survival (OS) of ER+ BC patients in association with the expression of the three pluripotency and ALDH1A1 genes.

Subgroup analyses revealed that some of these effects on OS could be dependent on PR and HER2 expression status.PR positivity appears to enhance the significant positive effect of ALDH1A1 on OS, as observed in the PR+ subgroup. None of the four markers exhibited a statistically significant effect in the ER+/PR− subgroup **(**Figure 3 **)**.

**FIGURE 4:**
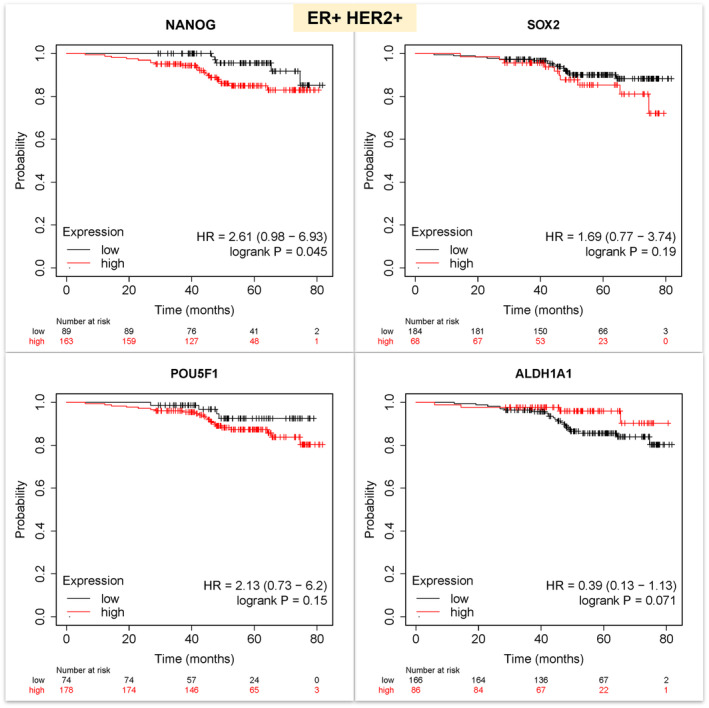
Kaplan–Meier survival analysis showing the effect of HER+ status on the overall survival (OS) of ER + BC patients in association with the expression of the three pluripotency and ALDH1A1 genes.

Meanwhile, in a subgroup of women with ER+ HER2+ BC (*n* = 252), only NANOG had a slightly significant unfavorable impact on OS (HR = 2.60, 95% CI: 0.98–6.90, *p* = 0.045). While SOX2 and OCT4 were linked to lower OS, the associations did not reach statistical significance. ALDH1A1 seemed to enhance survival, though not reaching statistical significance in the HER2+ subgroup (Figure 4).

#### 3.1.3 Mutational variants and effect on survival

**FIGURE 5:**
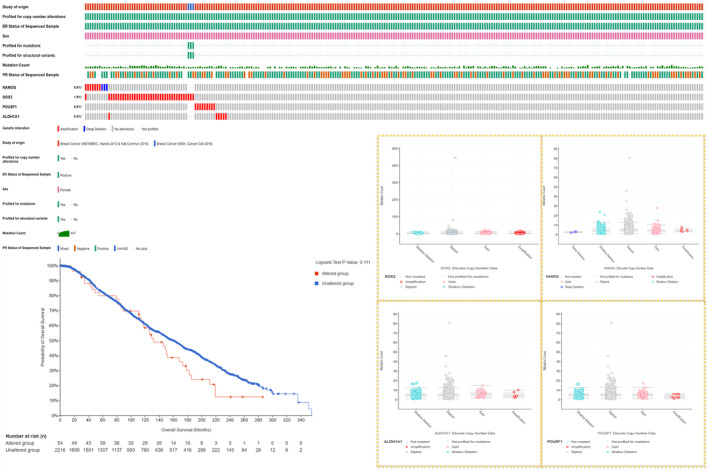
Analysis of copy number alterations (CNAs) and mutational variants in four genes (NANOG, SOX2, POU5F1, and ALDH1A1) among ER+ BC patients (*n* = 2607, including 10 studies, accessible at https://www.cbioportal.org/). The top panel displays the oncoprint for the selected genes and the corresponding CNAs, mutations, and clinical criteria. The left panel presents a comparison of overall survival between ER + BC patients with altered and unaltered genetic profiles, and the right panel illustrates scatter plots showing the distribution of CNAs in relation to mutation count within each one of the four genes.

Analysis of the cBioPortal datasets, encompassing 2607 ER + BC tumors from 10 studies, revealed that CNAs were most prevalent in SOX2, occurring in 1.6% of cases, predominantly as amplifications. NANOG and OCT4 exhibited similar CNA frequencies at 0.6%, and ALDH1A1 had a slightly lower frequency of 0.4%. Structural mutations in these genes were uncommon within the tumor samples. Furthermore, there was no notable difference in OS when comparing patients with gene alterations to those without, as illustrated in Figure 5.

### 3.2 Clinical study outcomes

The histopathologic tumor sections from a total of eighty‐one women with primary ER+ BC were eligible to be examined in this study. Clinical data including recognized risk factors are shown in Table 1. All women included in the study received antiestrogen medications (Tamoxifen or Letrozole) as per standard clinical protocols for adjuvant endocrine therapy for women with ER+ BC. The tumor sections were obtained at the time of primary surgery before the initiation of any therapy.

**TABLE 1:** Demographic and clinical data of study participants (*N* = 81).

Clinical factor	*N*	%
Age (years)		
<50	43	53.1
≥50	38	46.9
BMI (Kg/m^2^)		
<30	22	27.2
30–<35	34	42.0
≥35	25	30.9
Endocrine therapy		
Tamoxifen	48	59.3
Aromatase Inhibitor	33	40.7

In line with available evidence, this investigation included well‐established clinically recognized prognostic markers. These encompass clinical, molecular, and pathologic characteristics of the primary tumors as determined at the time of initial surgical excision (Table 2).

**TABLE 2:** Overview of the clinical, molecular, and pathologic prognostic indicators of the study subjects.

Prognostic factor^a^	*N*	%
Tumor size		
<20 mm	11	13.6
≥20– < 50 mm	41	50.6
≥**50 mm**	29	35.8
Clinical Stage		
Stages 1 and 2	39	48.1
**Stage 3**	42	51.9
Molecular class		
Luminal A	61	75.3
**Luminal B**	20	24.7
Ki‐67 index		
Low	66	81.5
**High**	15	18.5
HER2 expression		
Negative	73	90.1
**Positive**	8	9.9
Progesterone receptor expression		
Negative/Mild	29	35.8
Moderate/Strong	52	64.2
Nodal status		
N0	16	19.8
**N1/N2/N3**	65	80.2
Histological type		
Invasive lobular carcinoma	5	6.2
**Invasive ductal carcinoma**	72	88.9
Mixed	4	4.9
Histologic grade		
1	12	14.8
2	53	65.4
**3**	16	19.8
Perinodal fat		
Not infiltrated	39	48.1
**Infiltrated**	42	51.9
Lymphovascular invasion		
Not detected	31	38.3
**Detected**	50	61.7
In situ component		
Not detected	35	43.2
**Detected**	46	56.8

^a^ Bold outcomes are those with substantial evidence of their association with aggressive tumors or clinical outcomes. A few have heterogenous evidence on their prognostic value.

**FIGURE 6:**
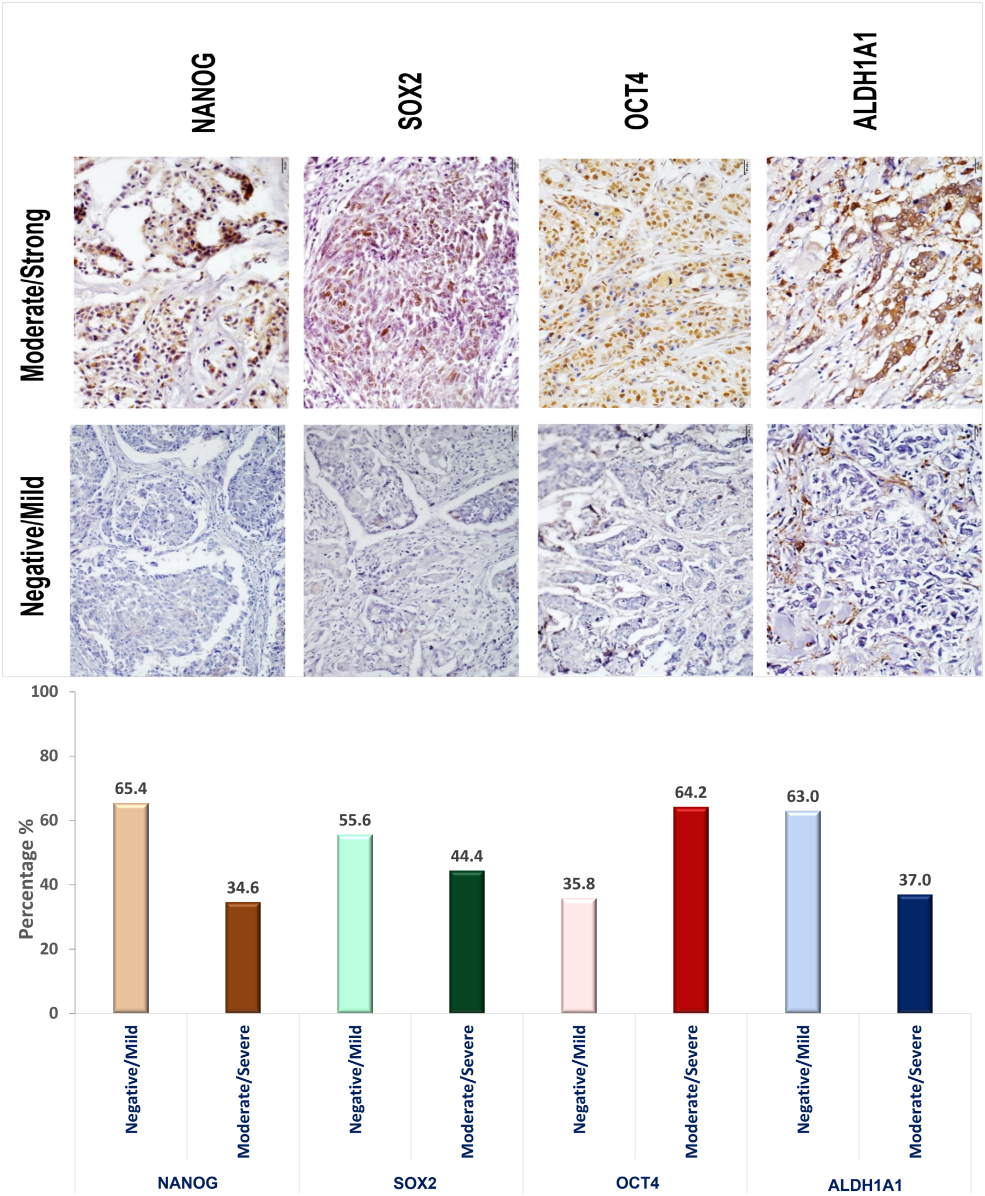
Bar chart illustrating the percent expression of the four stem cell markers examined in the study subjects (NANOG, SOX2, OCT4, and ALDH1A1). Insets at the top show representative immunohistochemical images (IHC 40×) illustrating the reference categories of marker expression applied in this study, distinguishing between negative/mild and moderate/strong expression. NANOG, SOX2, and OCT4 predominantly displayed nuclear expression, while ALDH1A1 exhibited cytoplasmic expression.

The prevalence and intensity of expression for the three master pluripotency markers (NANOG, SOX2, and OCT4) and ALDH1A1 in tumor tissue sections of the study participants is demonstrated in Figure 6, ranging from negative/mild to moderate/strong expressions. We identified substantial expression levels of NANOG, SOX2, OCT4, and ALDH1A1 in 34.6%, 44.4%, 64.2%, and 37% of the patients in our study cohort, respectively.

**FIGURE 7:**
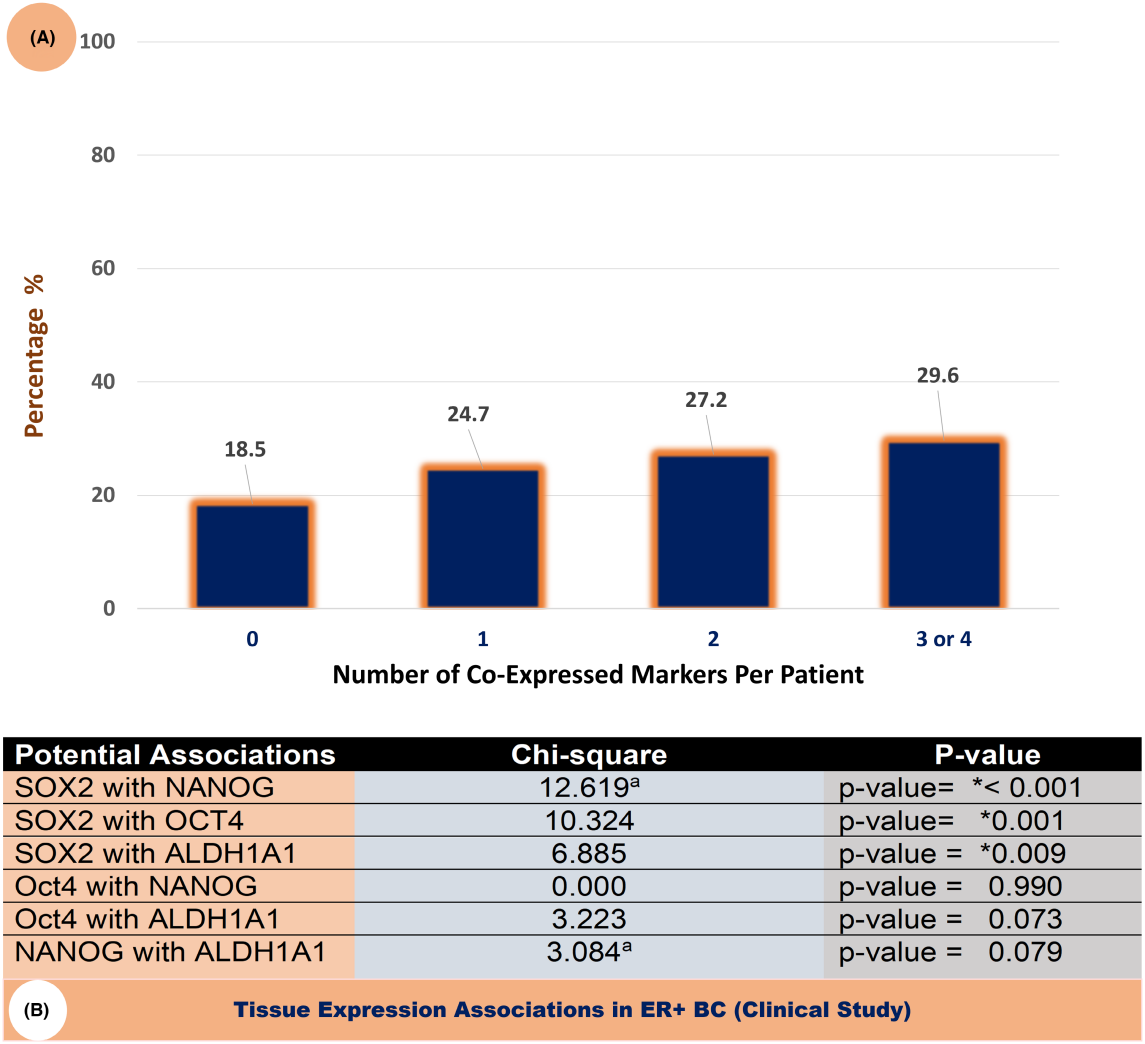
(A) Percent distribution of subjects by the number of markers per patient that were expressed at moderate or strong levels. (B) Tissue expression associations among the four markers using the chi‐squared test for the categories of expression as defined in this study (negative/mild, or moderate/strong).

Additionally, we examined if more than one of these markers were co‐expressed in the study subjects. Interestingly, 29.6% exhibited a moderate to strong expression of three or four markers, as shown in Figure 7A. In addition, to determine if there were synergistic or collaborative associations among the examined stem cell markers, we studied the co‐expression patterns between the individual markers. SOX2 exhibited a statistically significant difference in its tissue expression level (as indicated by the categories of expression outlined above) in association with the other three markers (NANOG, OCT4, and ALDH1A1 with *p*‐values of <0.001, 0.001, and 0.009 respectively), while there were no significant associations between NANOG, OCT4, and ALDH1A1 (Figure 7B).

To investigate potential associations between the stemness markers of interest and well‐established prognostic indicators, we examined the expression levels of each of the three pluripotency markers (SOX2, NANOG, and OCT4), as well as ALDH1A1 in relation to key clinical and molecular tumor prognostic factors (Table 3), as well as histopathologic factors (Table 4). It's important to note that for the sake of summarization, both tables only present prognostic factors associated with poor clinical outcomes, along with the chi‐squared and *p*‐values.

**TABLE 3:** Associations (percent expression %) of clinical and molecular indicators of poor prognosis by the degree of tissue expression of pluripotency markers and ALDH1A1 .

NANOG		SOX2		OCT4		ALDH1A1	
Negative/mild *N* = 53	Moderate/strong *N* = 28	Negative/mild *N* = 45	Moderate/strong *N* = 36	Negative/mild *N* = 29	Moderate/strong *N* = 52	Negative/mild *N* = 51	Moderate/strong *N* = 30
*n* (%)	*n* (%)	*n* (%)	*n* (%)	*n* (%)	*n* (%)	*n* (%)	*n* (%)
Large tumor size >5 cm							
21 (39.6%)	8 (28.6%)	15 (33.3%)	14 (38.9%)	11 (37.9%)	18 (34.6%)	19 (37.3%)	10 (33.3%)
Chi‐square = 1.746 *p*‐value = 0.418		Chi‐square = 0.325 *p*‐value = 0.850		Chi‐square = 0.416 *p*‐value = 0.812		Chi‐square = 0.875 *p*‐value = 0.646	
Stage 3 (locally advanced tumor)							
27 (50.9%)	15 (53.6%)	20 (44.4%)	22 (61.1%)	14 (48.3%)	28 (53.8%)	27 (52.9%)	15 (50.0%)
Chi‐square = 0.051 *p*‐value = 0.822		Chi‐square = 2.225 *p*‐value = 0.136		Chi‐square = 0.231 *p*‐value = 0.631		Chi‐square = 0.065 *p*‐value = 0.798	
Molecular class luminal B							
13 (24.5%)	7 (25.0%)	9 (20.0%)	11 (30.6%)	5 (17.2%)	15 (28.8%)	9 (17.6%)	11 (36.7%)
Chi‐square = 0.002 *p*‐value = 0.963		Chi‐square = 1.198 *p*‐value = 0.274		Chi‐square = 1.348 *p*‐value = 0.246		Chi‐square = 3.675 *p*‐value = 0.055	
High Ki‐67							
9 (17.0%)	6 (21.4%)	5 (11.1%)	10 (27.8%)	1 (3.4%)	14 (26.9%)	5 (9.8%)	10 (33.3%)
Chi‐square = 0.240 *p*‐value = 0.624		Chi‐square = 3.682 *p*‐value = 0.055		Chi‐square = 6.799 *p*‐value = *0.009		Chi‐square = 6.930 *p*‐value = *0.008	
HER2‐positive disease							
7 (13.2%)	1 (3.6%)	5 (11.1%)	3 (8.3%)	3 (10.3%)	5 (9.6%)	3 (5.9%)	5 (16.7%)
Chi‐square = 1.911 *p*‐value = 0.167		Chi‐square = 0.173 *p*‐value = 0.677		Chi‐square = 0.011 *p*‐value = 0.916		Chi‐square = 2.468 *p*‐value = 0.116	

Bold indicates significance level at *p* value <0.05.

**TABLE 4:** Associations (percent expression %) of histopathologic predictors of poor outcome by the degree of tissue expression of pluripotency markers and ALDH1A1.

	NANOG		SOX2		OCT4		ALDH1A1	
	Negative/mild *N* = 53	Moderate/strong *N* = 28	Negative/Mild *N* = 45	Moderate/strong *N* = 36	Negative/mild *N* = 29	Moderate/strong *N* = 52	Negative/mild *N* = 51	Moderate/strong *N* = 30
	*n* (%)	*n* (%)	*n* (%)	*n* (%)	*n* (%)	*n* (%)	*n* (%)	*n* (%)
Histologic grade								
Grade 3	9 (17.0%)	7 (25.0%)	9 (20.0%)	7 (19.4%)	6 (20.7%)	10 (19.2%)	11 (21.6%)	5 (16.7%)
	Chi‐square = 0.750 *p*‐value = 0.687		Chi‐square = 0.763 *p*‐value = 0.683		Chi‐square = 0.278 *p*‐value = 0.870		Chi‐square = 3.284 *p*‐value = 0.194	
Perinodal fat infiltration								
Infiltrated	29 (54.7%)	13 (46.4%)	18 (40.0%)	24 (66.7%)	13 (44.8%)	29 (55.8%)	26 (51.0%)	16 (53.3%)
	Chi‐square = 0.504 *p*‐value = 0.478		Chi‐square = 5.697 *p*‐value = *0.017		Chi‐square = 0.893 *p*‐value = 0.345		Chi‐square = 0.042 *p*‐value = 0.838	
Lymphovascular invasion								
Detected	32 (60.4%)	18 (64.3%)	25 (55.6%)	25 (69.4%)	18 (62.1%)	32 (61.5%)	31 (60.8%)	19 (63.3%)
	Chi‐square = 0.118 *p*‐value = 0.731		Chi‐square = 1.633 *p*‐value = 0.201		Chi‐square = 0.002 *p*‐value = 0.962		Chi‐square = 0.052 *p*‐value = 0.820	
Nodal status								
N1/N2/N3	44 (83.0%)	21 (75.0%)	34 (75.6%)	31 (86.1%)	22 (75.9%)	43 (82.7%)	42 (82.4%)	23 (76.7%)
	Chi‐square = 0.743 *p*‐value = 0.389		Chi‐square = 1.406 *p*‐value = 0.236		Chi‐square = 0.548 *p*‐value = 0.459		Chi‐square = 0.385 *p*‐value = 0.535	

Bold indicates significance level at *p* value <0.05.

For tumor expression of the progesterone receptor (PR), only OCT4—among all the four markers‐ was found to be significantly associated with the expression of the PR (*p‐*value *= 0.026*).

## 4 DISCUSSION

In this study, we examined the expression of the pluripotency genes (NANOG, SOX2, and POU5F1) and the ALDH1A1 gene in ER+ BC tumors across three large cancer datasets. We aimed to identify relationships between gene expression levels and patient survival outcomes. We also explored correlations and potential interactions among these markers and with established prognostic factors. Additionally, we assessed the expression of the same marker panel‐using IHC studies‐ in tumor sections from eighty‐one women with primary ER+ BC. Within this cohort, we determined marker expression levels, explored co‐expression patterns, and investigated associations with established clinical, pathologic, and molecular prognostic markers. Unlike the substantial evidence for the role of cancer stem cells (CSCs) and their markers in ER‐negative BC, the present study contributes new data to the limited literature on the potential role of stem cell markers in ER+ BC. It also provides insights into possible interactions as well as differences among these individual markers.

In the clinical study cohort, the assessment of protein expression using IHC revealed moderate to strong expression of all four stem cell markers in at least one‐third of the subjects. Additionally, more than half of the tumors (56.8%) exhibited moderate to strong co‐expression of two or more of these markers (refer to Figures 5 and 6). These observations align with the gene expression data obtained from two distinct cancer datasets, namely KM‐plotter and GENT2. As illustrated in Figure 2, ER+ BC patients who were included in the KM survival analysis displayed high expression levels, with NANOG at around 66%, SOX2 at 54%, POU5F1 at 31%, and ALDH1A1 at 44%. This is also consistent with the findings from GENT2 analysis (Figure 1), which demonstrated significant expression of NANOG and ALDH1A1 in ER+ BC compared to ER−BC patients. Taken together, the findings emphasize the substantial expression of these stem cell markers in ER + BC, demanding the need for attention and further investigations.

In the gene expression/survival study utilizing RNA‐Sequence data of ER+ BC patients who received endocrine therapy, we identified a statistically significant association between the high expression of NANOG and poor overall survival (OS) of ER+ BC patients, particularly prominent in the subgroup of patients with HER2+ status. SOX2 and POU5F1 (OCT4 gene) expression did not have a statistically significant effect on OS. In contrast, an intriguing finding in this study is the significant association between high ALDH1A1 expression and improved OS in ER+ BC patients, particularly within the PR+ subgroup. However, this advantage was not statistically significant in both the PR− and HER2+ subgroups.

In general, these findings deviate from the prevailing evidence linking ALDH1A1 overexpression to aggressive tumor behavior and poor prognosis, a connection often justified by the ability of ALDH‐positive cancer cells to form colonies with high tumorigenic potential.^29^, ^30^ Notably, much of this evidence was derived from investigations of ER‐negative or/triple‐negative BC,^29^, ^31^ considered the most aggressive and most linked to CSC activity. Other evidence, based on studies with heterogeneous receptor status, suggests ALDH1A1 as a biomarker predicting tumor progression and poor survival in BC patients.^32^ ALDH1A1 was also shown as a more relevant marker for CSC‐mediated resistance compared to other breast CSC markers, such as CD44 high/CD24 low.^33^ The focus on ER‐negative tumors is also supported by data indicating ALDH1A1 positive cells are significantly more likely to be ER− and HER+.^34^ Moreover, since aldehyde dehydrogenase enzymes can efficiently detoxify various substrates by oxidizing them into carboxylic acids,^35^ this mechanism has been widely adopted to explain tumor resistance to cytotoxic chemotherapies. Therefore, the overexpression of the ALDH1A1 gene and the increased enzyme activity have been thought to be crucial for CSCs to resist chemotherapy. This is particularly relevant in ER‐negative tumors, which are almost exclusively treated with cytotoxic therapies.^35^ As a result, this rationale directed the search for therapeutic molecular targets that can inhibit the expression of ALDH1A1 in ER‐negative BC.^36^

Nonetheless, a growing body of evidence suggests that the role of ALDH1A1 is more complex than previously thought. Such complexity can be understood considering the recognized physiological cellular roles of ALDH1 isoenzymes, coupled with evidence from several other cancers where ALDH1A1 upregulation was linked to well‐differentiated tumors and favorable prognostic outcomes.^35^, ^37^, ^38^ Liu Yan et al. further challenged the dominant view by demonstrating that high ALDH1A1 mRNA expression in triple‐negative BC is associated with improved survival.^39^ Hence, there is a reasonable possibility that ALDH1A1 expression in ER+ BC may play a beneficial prognostic role, which requires further prospective investigations to be confirmed. Nevertheless, methodological heterogeneity should be considered and could contribute to the inconsistency between poor and favorable outcomes in various BC studies. This includes factors related to tumor and patient selection, as well as the cut‐off for ALDH1A1 overexpression.^40^

Additionally, we examined the possible inter‐associations and correlations among the four stem cell markers in the clinical and the in‐silico studies. In the clinical cohort, we observed that the degree of cellular expression of SOX2 was highly associated with the degree of expression of each of the three other markers (NANOG, OCT4, and ALDH1A1), as shown in Figure 7. However, no significant co‐expression associations were found among the other markers. Analyzing the quantitative gene expression RNA‐Seq data, as illustrated in Figure 2, we observed three statistically significant correlations. Among them, the most notable is a weak positive correlation between SOX2 and ALDH1A1, with a correlation coefficient of 0.11 (*p* < 1E−04). Previous studies suggested a possibility of collaborative relationships among the pluripotency markers. For instance, SOX2‐OCT4 composites were found to co‐bind DNA, and that binding was critical to inducing and maintaining the pluripotency of CSCs.^41^ Likewise, the Co‐expression of OCT4 and NANOG has been linked to reduced survival and a more aggressive BC behavior. This association was attributed to the activation of the epithelial‐mesenchymal transition (EMT) phenotype, as demonstrated in a study involving 126 BC patients, predominantly with ER+ tumors. This finding aligns, in part, with the observed impact of NANOG in our gene expression analysis additionally, the same study showed evidence that NANOG and OCT4 may mutually induce each other's expression.^42^

In the examined tumor sections of women with ER + BC, various clinical and molecular prognostic markers such as clinical stage, tumor size, molecular class, HER2 status, Ki‐67 index, and PR status, were investigated. Notably, the moderate/strong expression of OCT‐4 or ALDH1A1 was significantly associated with a high Ki‐67 index (as indicated in Table 3). Given this observed categorical association, we examined the quantitative correlation between ALDH1A1 and Ki‐67 across different ER + BC subtypes and molecular profiles using the KM‐plotter dataset, and their independent effect on survival outcomes using the GENT2 tool. The analysis revealed a mild negative correlation which was statistically significant within the specified ER+ BC group (refer to the detailed Data S1). However, due to the lack of complete survival data in the clinical study and the anticipated methodological differences between IHC‐based marker assessment and RNA‐Seq gene expression data, direct comparisons of these findings are not accurate. However, the examined datasets support the association of Ki‐67 with poor survival across the various subtypes and molecular profiles, aligning with existing evidence which indicates that Ki‐67 serves as a cell proliferation marker, and its high expression is linked to unfavorable survival outcomes.^43^

The effect of the POU5F1 gene and its marker product (OCT4) was inconclusive between the RNA‐Seq analysis and the IHC results. In a relevant study by Gwak JM et al, it was identified as a crucial prognostic factor linked to ALDH1 expression and a poor response to tamoxifen in 221 ER+ BC patients.^44^ In addition, our IHC study findings showed that moderate/strong tissue expression of OCT‐4 was significantly linked to progesterone receptor (PR) positivity. While mainstream evidence suggests that ER+/PR+ BC patients generally exhibit a favorable prognosis than ER+/PR− cases, the role of PR in predicting outcomes remains complex and inconsistently predictive.^45^ One previous small study has found no significant correlation between OCT‐4 and other tumor phenotypes including PR status.^46^

Moreover, for key associations with histopathologic prognostic markers, perinodal fat invasion by cancer cells was significantly predicted by the moderate/strong expression of SOX2. The extension of cancer cells through the axillary lymph node capsule into the perinodal fat was previously found to be an independent prognostic factor, predictive of elevated risk of mortality and recurrence.^47^ Based on the IHC study's findings, SOX2 was associated with perinodal fat invasion. SOX2 has been recognized for its oncogenic role in BC, and its involvement in the mammosphere formation capacity of BC cells, a key functional feature of CSCs.^48^, ^49^ Furthermore, recent research has highlighted SOX2 as a consistent marker for tumor‐initiating cells (TICs) in ovarian cancer contributing to relapse and resistance to chemotherapy.^50^ Additionally, Piva et al. identified an association between SOX2 overexpression and BC cell resistance to tamoxifen, a resistance that can be reversed by knocking out SOX2.^51^

Our study has limitations, notably the relatively small number of examined tumors in the clinical IHC study. However, this limitation is partially mitigated by the selective inclusion criteria, which ensured that the patient characteristics and profiles of the clinical study closely resembled those from the investigated three large cancer datasets encompassing rich clinical, molecular, and genomic data. Together, this approach has yielded new insights into the potential prognostic value of these markers in the context of ER+ BC.

## 5 CONCLUSION

In this study, we present evidence regarding the role of the three master embryonic pluripotency markers (NANOG, SOX2, and OCT4) along with the stem cell marker ALDH1A1 in estrogen receptor (ER) positive tumors. Our research incorporates data from gene expression survival analysis using three key cancer datasets, along with immunohistochemical studies conducted on a comparable cohort of ER+ BC patients. The survival analysis studies demonstrated the significant impact of these markers on the survival of ER+ BC patients. Moreover, their expression in the cohort of ER+ BC patients was substantial in terms of both frequency and degree, with significant associations with established clinicopathologic prognostic indicators, such as Ki‐67, PR, and perinodal fat invasion. Importantly, our findings have not only shown significant associations, but have also shed light on distinct roles among these individual pluripotency markers. The data derived from this study may hold implications for new prognostic biomarkers as well as therapeutic targets in women affected by ER + BC.

## AUTHOR CONTRIBUTIONS

**Noha A. Mousa:** Conceptualization (equal); funding acquisition (equal); methodology (equal); supervision (equal); writing – original draft (equal); writing – review and editing (equal). **Amal Hussein:** Data curation (equal); formal analysis (equal); writing – review and editing (equal). **Noha M. Elemam:** Investigation (equal); writing – review and editing (equal). **Ghada Mohammed:** Conceptualization (equal). **Mona Elwany:** Data curation (equal); investigation (equal); validation (equal). **Tasneem Basha:** Data curation (equal); validation (equal). **Amal A. Alhammadi:** Data curation (equal); writing – review and editing (equal). **Rana S. Majzob:** Investigation (equal); validation (equal). **Iman M. Talaat:** Investigation (equal); methodology (equal); validation (equal); writing – review and editing (equal).

## FUNDING INFORMATION

The project was funded by the University of Sharjah (Competitive Grant # 1901090266).

## CONFLICT OF INTEREST STATEMENT

All authors declare no conflict of interest in this study.

## ETHICS STATEMENT

Ethical approval of the study was obtained from the Research Ethics Committee (REC) of the University of Sharjah (REC‐20‐02‐03‐01). The written informed consent was waived by the REC, as this study was retrospective and involved the retrospective use of anonymous FFPE blocks.

## Supporting information


Data S1:

## Data Availability

Data obtained from open datasets are available online as adequately cited in the references. All original data sheets of the clinical study and statistical analysis data are available with the corresponding authors and will be provided upon reasonable request.
