# Correction to “Are embryonic stem cell markers and ALDH1A1 relevant in the context of breast cancer estrogen positivity”

**DOI:** 10.1002/cam4.7171

**Published:** 2024-04-17

Mousa NA, Hussein A, Elemam NM, et al. Are embryonic stem cell markers and ALDH1A1 relevant in the context of breast cancer estrogen positivity? Cancer Med. 2024;13:e7004. doi:10.1002/cam4.7004

The affiliations of Iman M. Talaat shown below have been added to the online version of the article.

**Iman M. Talaat** ^**1,3,5**^

^1^Clinical Sciences Department, College of Medicine, University of Sharjah, Sharjah, United Arab Emirates

^2^Family and Community Medicine and Behavioral Sciences Department, College of Medicine, University of Sharjah, Sharjah, United Arab Emirates

^3^Research Institute for Medical and. Health Sciences, University of Sharjah, Sharjah, United Arab Emirates

^4^Medical Research Institute, Alexandria University, Alexandria, Egypt

^5^Pathology Department, Faculty of Medicine, Alexandria University, Alexandria, Egypt

