# Supplementary material for: Are embryonic stem cell markers and ALDH1A1 relevant in the context of breast cancer estrogen positivity?
Source: Cancer Med. 2024 Feb 24;13(3):e7004. doi: 10.1002/cam4.7004 (PMC10891463; doi:10.1002/cam4.7004)
Supplement: Supplementary file 1 — Data S1: [file CAM4-13-e7004-s001.pptx]

## Slide 1
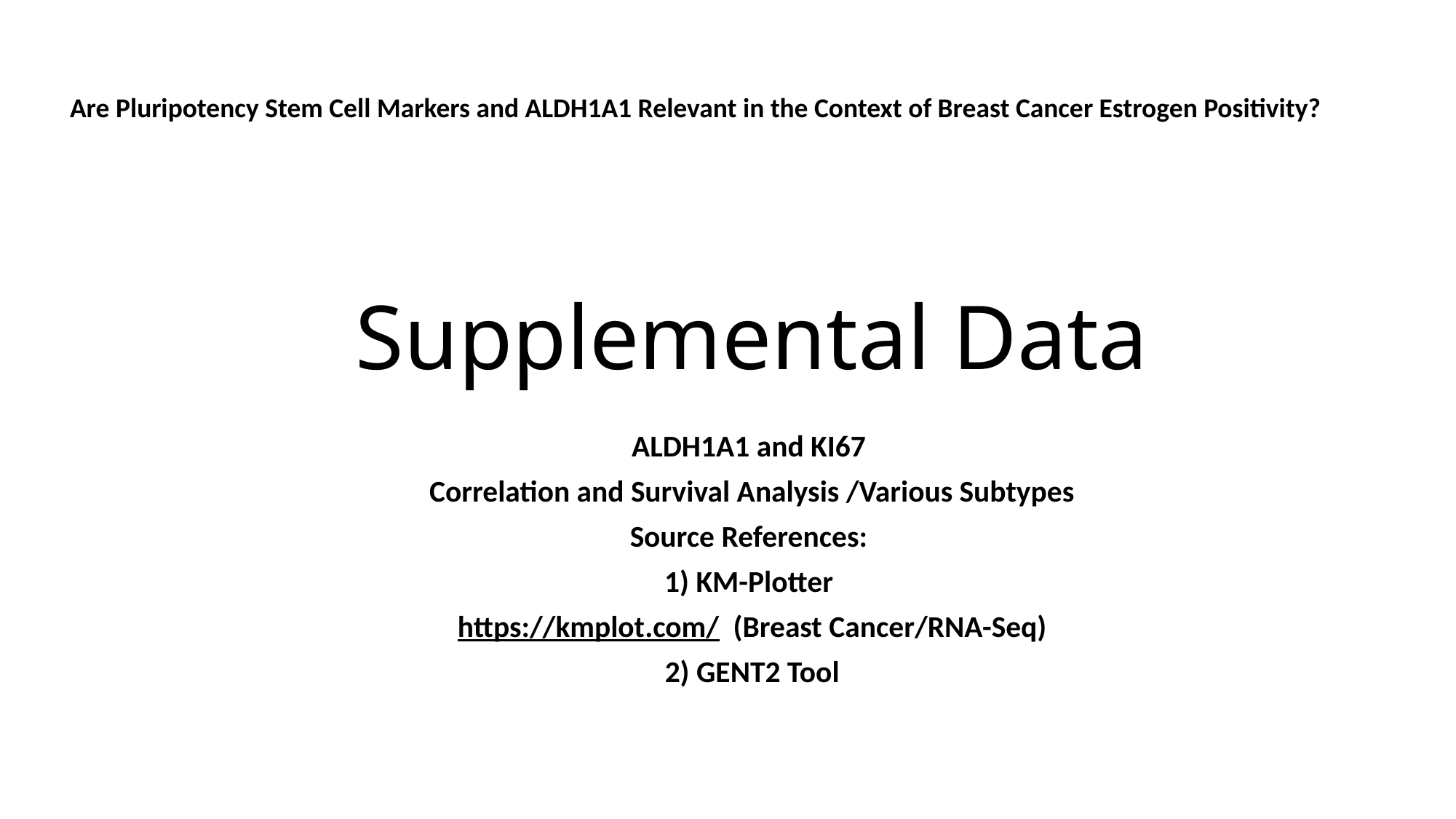

Are Pluripotency Stem Cell Markers and ALDH1A1 Relevant in the Context of Breast Cancer Estrogen Positivity?
# Supplemental Data
ALDH1A1 and KI67
Correlation and Survival Analysis /Various Subtypes
Source References:
1) KM-Plotter
https://kmplot.com/ (Breast Cancer/RNA-Seq)
2) GENT2 Tool

## Slide 2
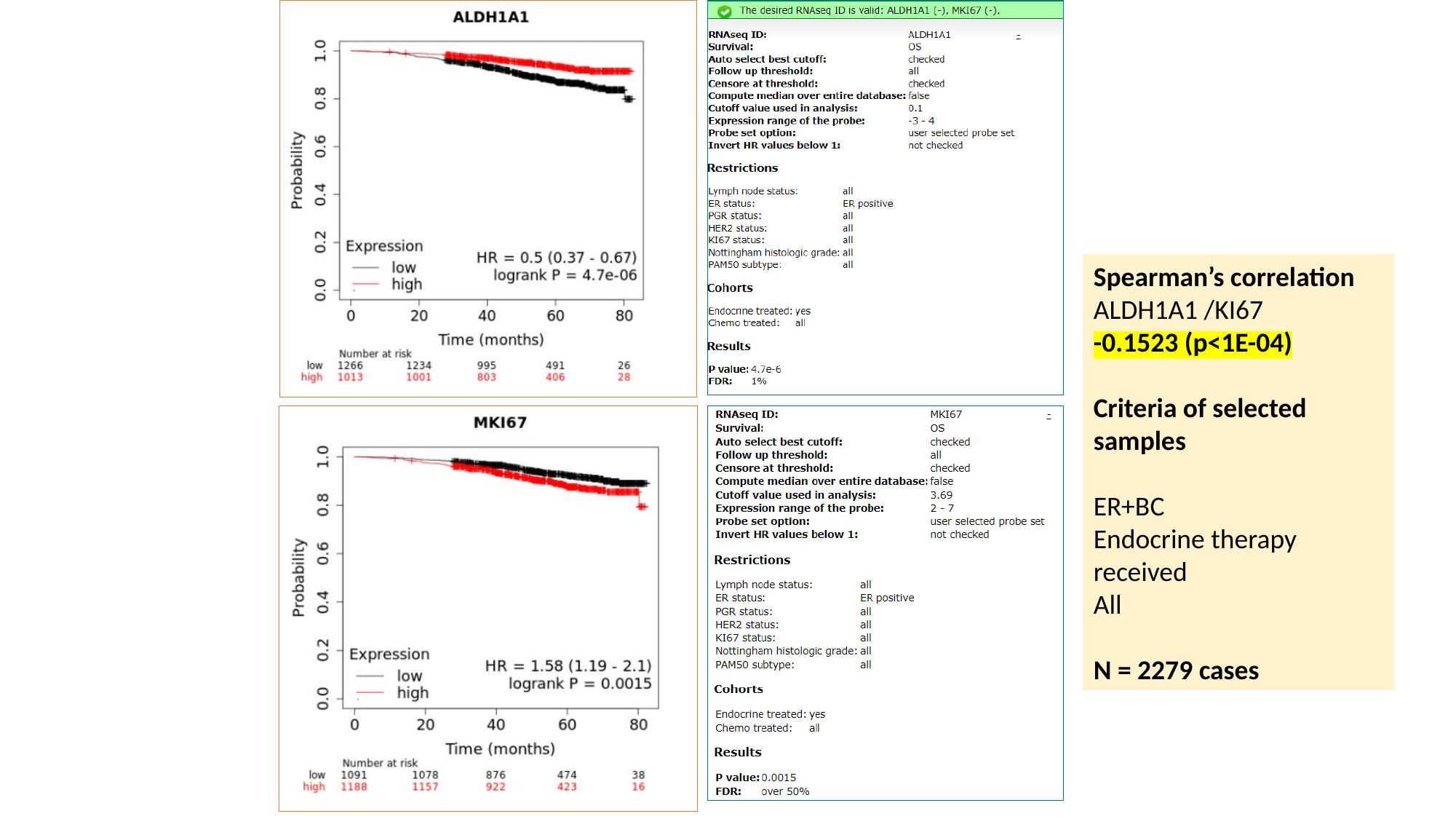

Spearman’s correlation ALDH1A1 /KI67
-0.1523 (p<1E-04)
Criteria of selected samples
ER+BC
Endocrine therapy received
All
N = 2279 cases

## Slide 3
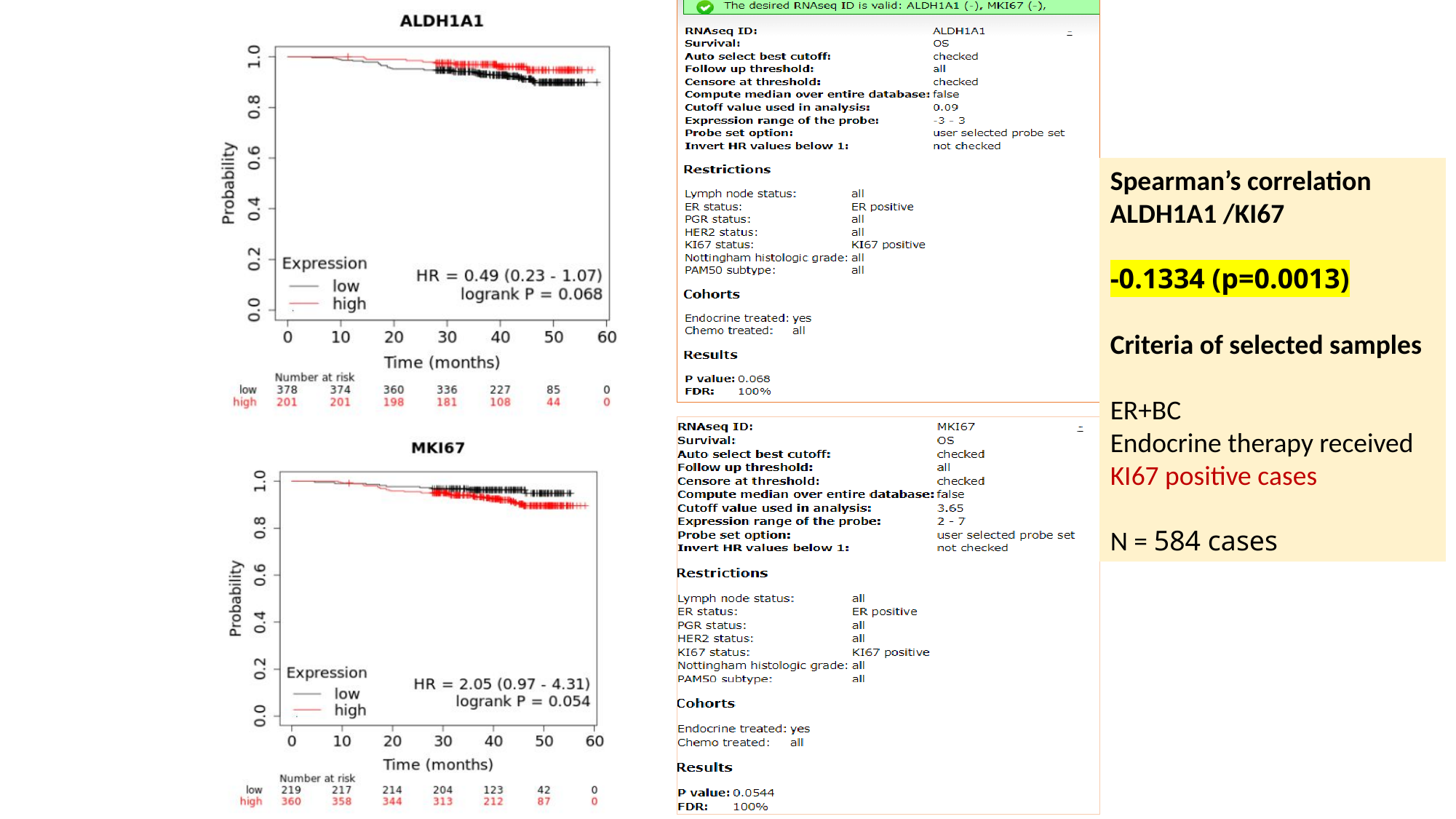

Spearman’s correlation ALDH1A1 /KI67
-0.1334 (p=0.0013)
Criteria of selected samples
ER+BC
Endocrine therapy received
KI67 positive cases
N = 584 cases

## Slide 4
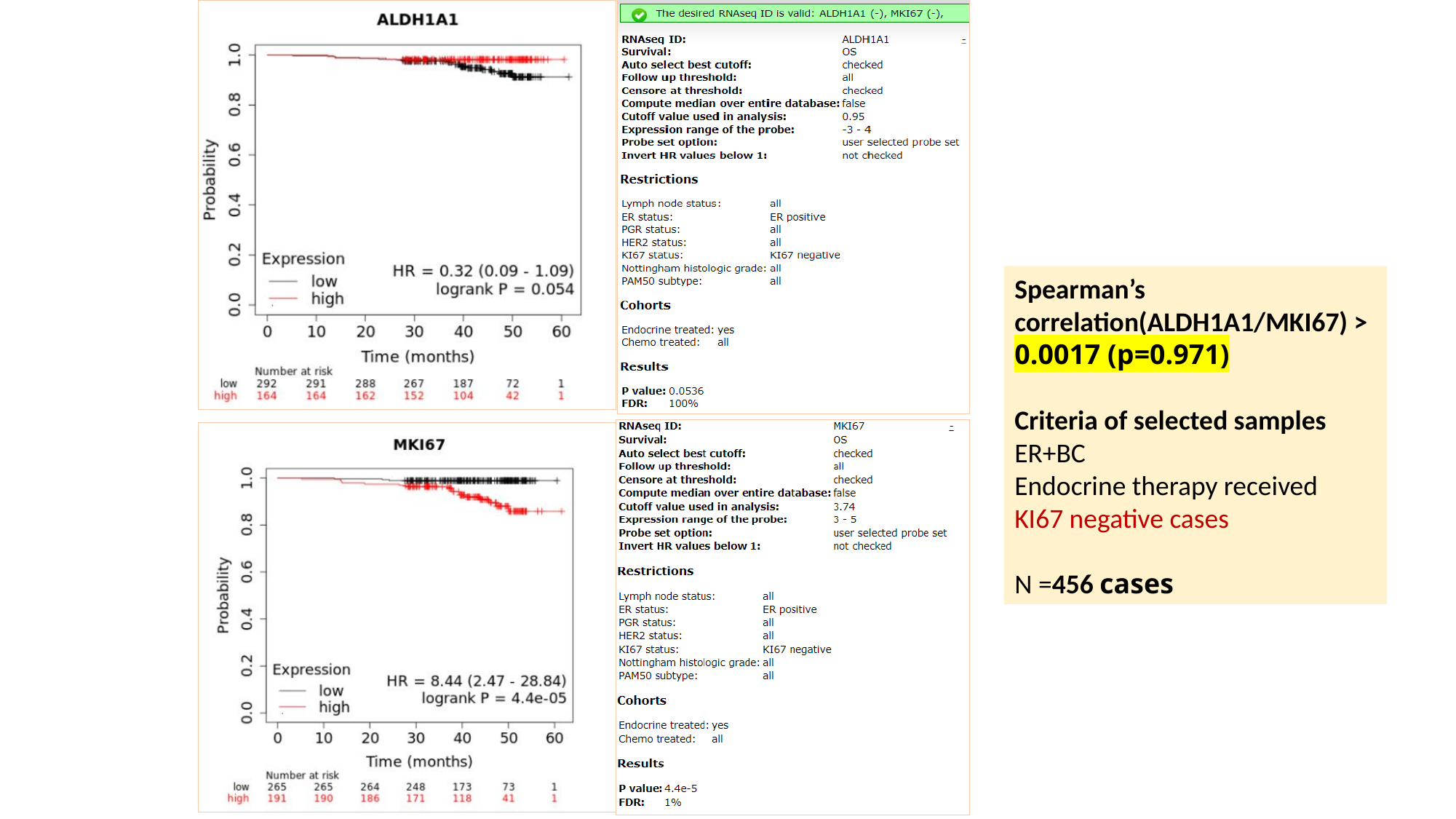

Spearman’s correlation(ALDH1A1/MKI67) > 0.0017 (p=0.971)
Criteria of selected samples
ER+BC
Endocrine therapy received
KI67 negative cases
N =456 cases

## Slide 5
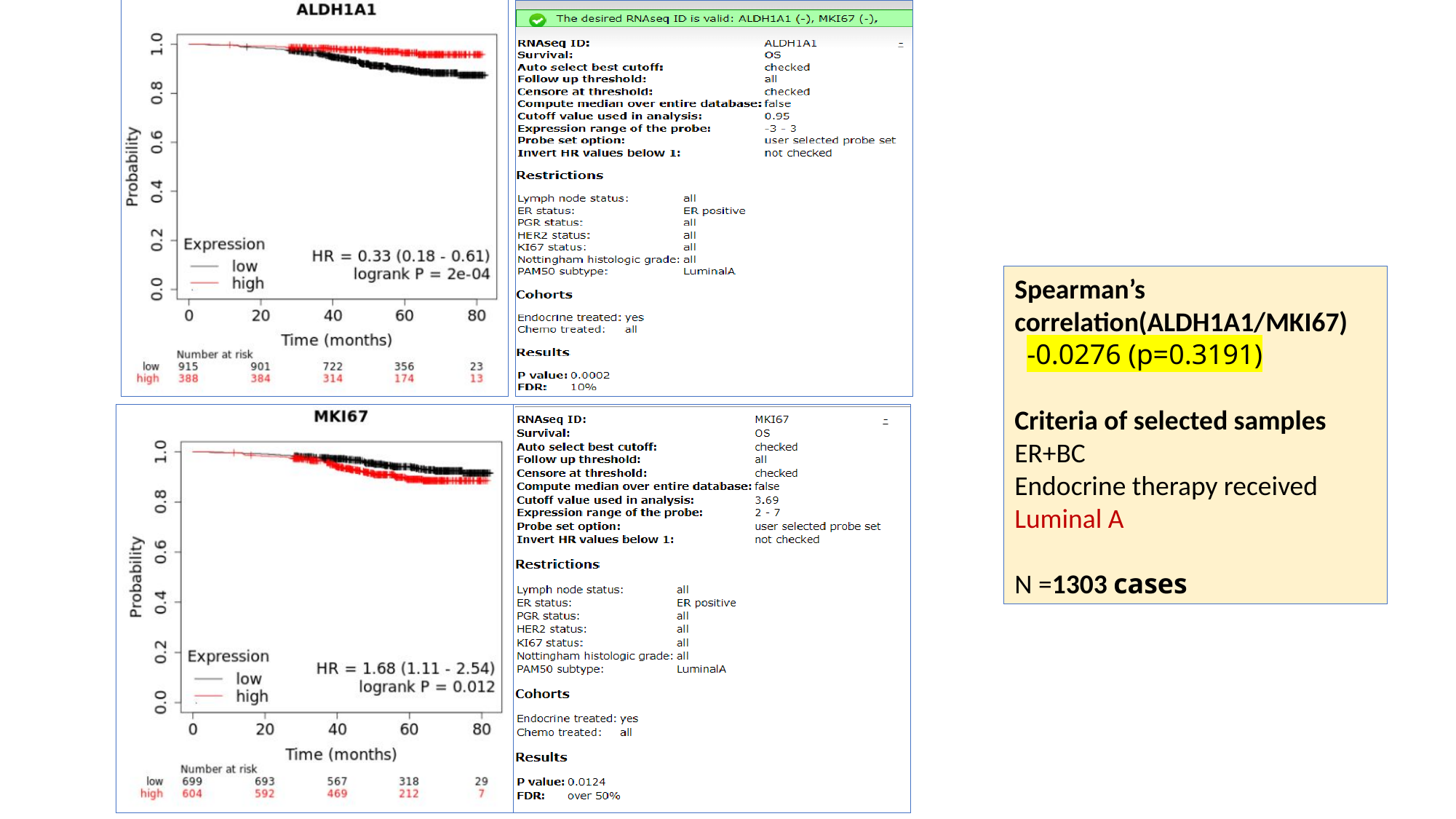

Spearman’s correlation(ALDH1A1/MKI67)
 -0.0276 (p=0.3191)
Criteria of selected samples
ER+BC
Endocrine therapy received
Luminal A
N =1303 cases

## Slide 6
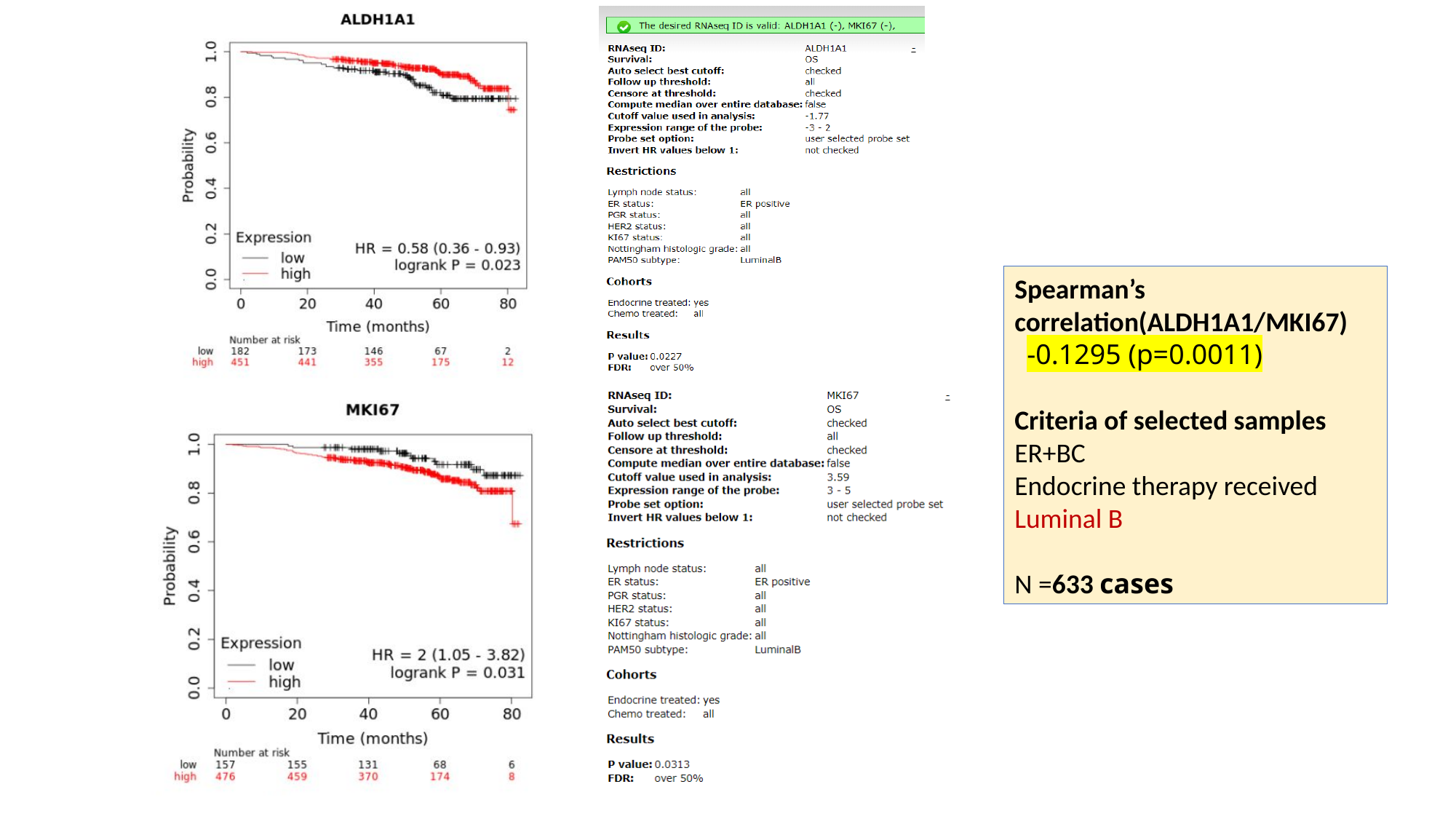

Spearman’s correlation(ALDH1A1/MKI67)
 -0.1295 (p=0.0011)
Criteria of selected samples
ER+BC
Endocrine therapy received
Luminal B
N =633 cases

## Slide 7
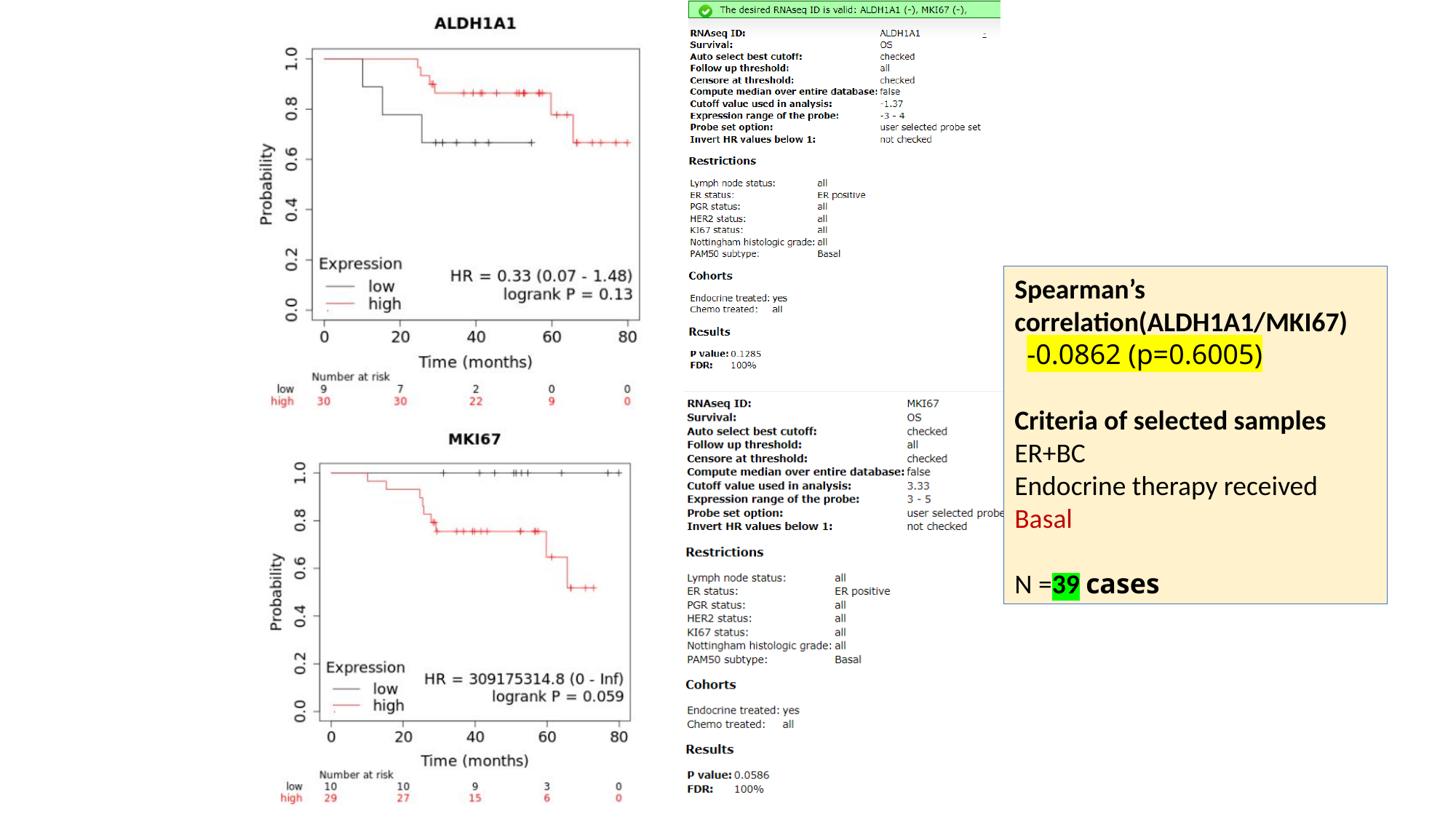

Spearman’s correlation(ALDH1A1/MKI67)
 -0.0862 (p=0.6005)
Criteria of selected samples
ER+BC
Endocrine therapy received
Basal
N =39 cases

## Slide 8
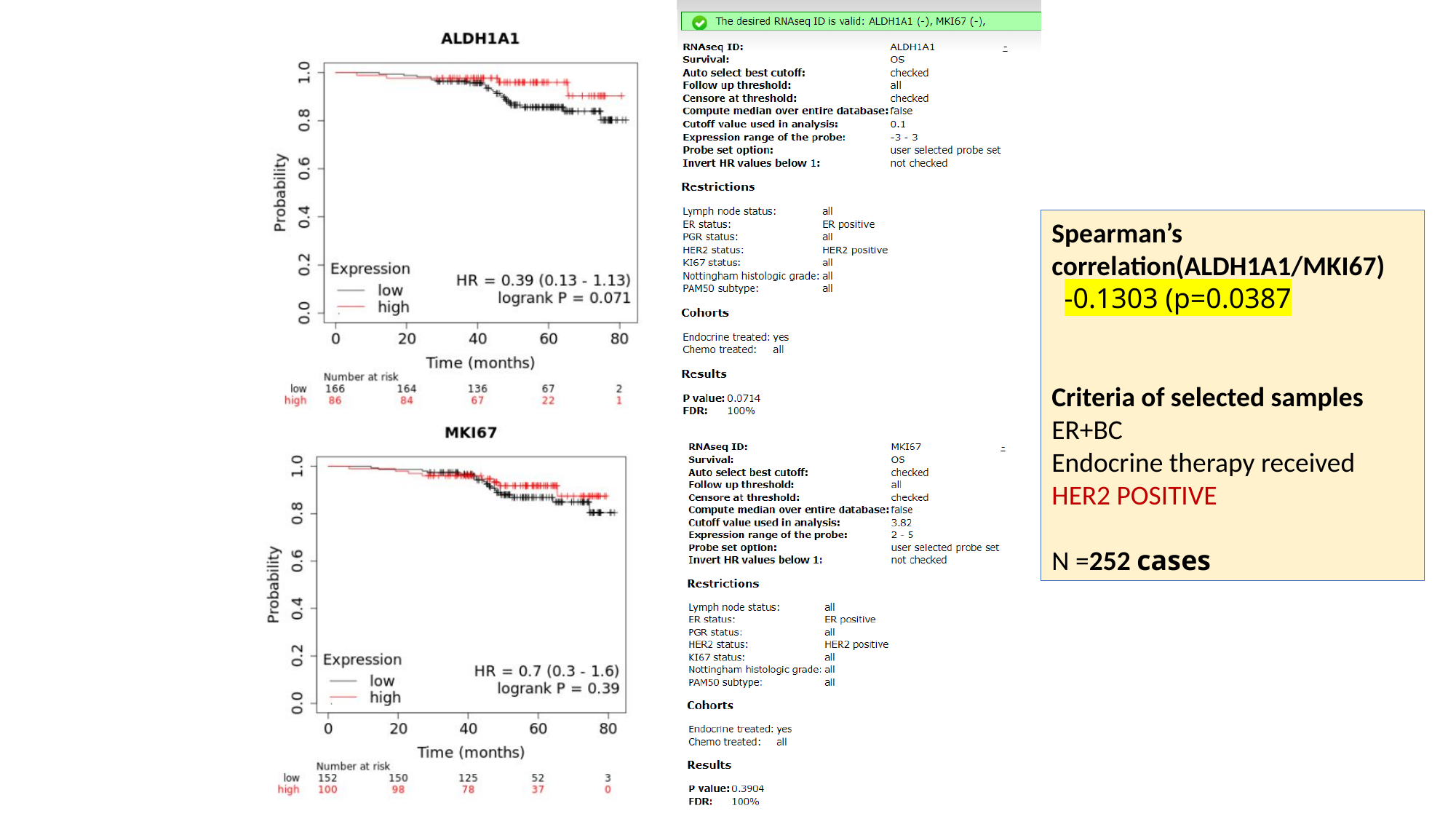

Spearman’s correlation(ALDH1A1/MKI67)
 -0.1303 (p=0.0387
Criteria of selected samples
ER+BC
Endocrine therapy received
HER2 POSITIVE
N =252 cases

## Slide 9
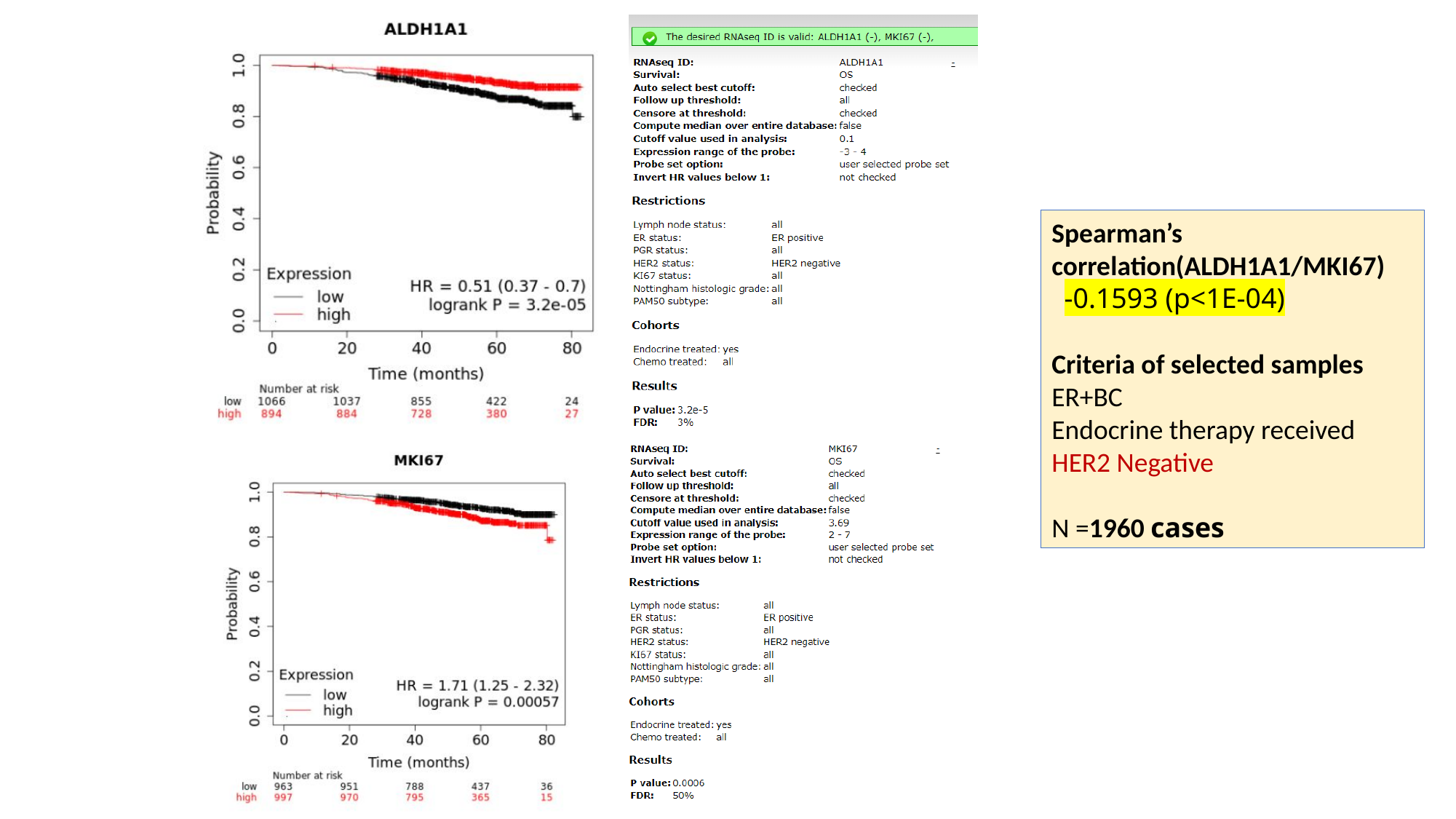

Spearman’s correlation(ALDH1A1/MKI67)
 -0.1593 (p<1E-04)
Criteria of selected samples
ER+BC
Endocrine therapy received
HER2 Negative
N =1960 cases

## Slide 10
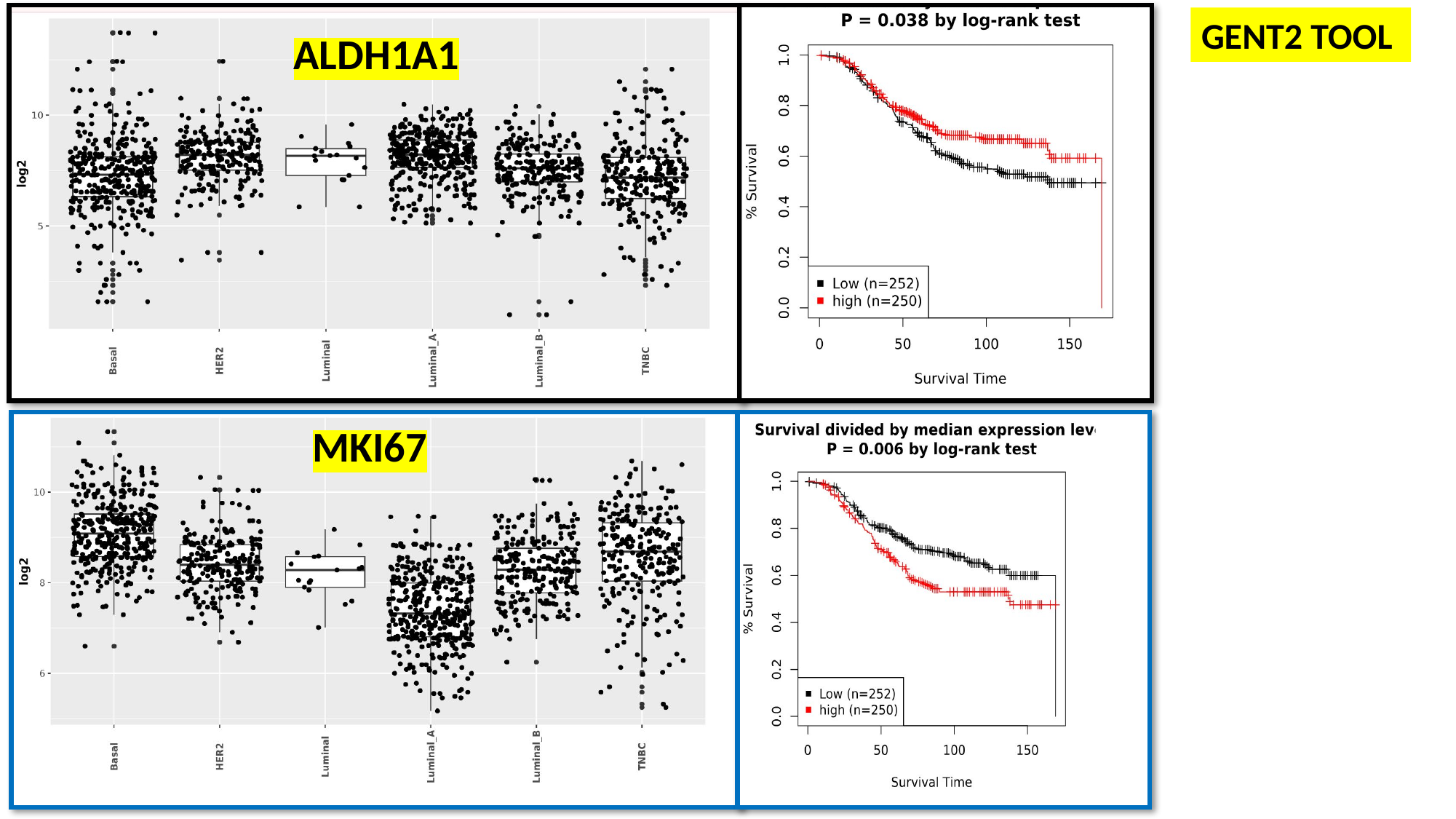

ALDH1A1
MKI67
GENT2 TOOL
